# Extracranial Meningioma En Plaque With Skull Invasion

**DOI:** 10.7759/cureus.50490

**Published:** 2023-12-13

**Authors:** Corneliu Toader, Razvan-Adrian Covache-Busuioc, Bogdan-Gabriel Bratu, David-Ioan Dumitrascu, Matei Serban, Alexandru Vladimir Ciurea

**Affiliations:** 1 Department of Neurosurgery, Carol Davila University of Medicine and Pharmacy, Bucharest, ROU; 2 Department of Vascular Neurosurgery, National Institute of Neurology and Neurovascular Diseases, Bucharest, ROU; 3 Department of Neurosurgery, Sanador Clinical Hospital, Bucharest, ROU

**Keywords:** gender disparity in incidence, age-related susceptibility, surgical approaches, world health organization grades, histological classification, postoperative complications, osteolytic changes, gross total resection, extracranial meningiomas, meningioma en plaque

## Abstract

The study reflects on a 69-year-old female patient with a history of cardio-respiratory disorders who was diagnosed with meningioma en plaque. Her clinical management entailed surgical resection of the tumor, which was followed by a complex postoperative course, including cardiorespiratory arrest and respiratory failure. Histologically, extracranial meningiomas are categorized into five subtypes based on predominant cellular morphology, with the meningothelial type being prevalent in this case. The report also examines the significance of complete tumor resection, noting a lower recurrence rate with gross total resection. Additionally, it discusses the increased susceptibility of extracranial meningiomas with advancing age and a higher incidence in females. Data from various studies underscore the importance of a surgical approach and extent of resection in predicting recurrence risk. The case report concludes by highlighting the critical aspects of the pathology of meningiomas and the surgical strategy that ensured the patient's recovery. The findings from this case contribute to the broader understanding of extracranial meningiomas, their diagnosis, and management.

## Introduction

Meningiomas are slow-growing neoplasms carrying a favorable prognosis, especially when entirely resected and in younger patients [1]. Accounting for 15-20% of all brain tumors, they rarely extend beyond the cranial boundaries and usually associate osteolytic changes in the skull [2]. Primary extracranial meningiomas are uncommon tumors that are often misdiagnosed and are located in a broad range of sites, with approximately 6-17% of all meningiomas being found in extranevraxial locations. Thus, recognizing this diagnosis in unexpected spots could prevent potential challenges [3]. Typically, meningiomas are grouped into three grades, based on the World Health Organization (WHO) Meningioma Classification as follows: grade I (benign), grade II (atypical), and grade III (malignant), with the benign ones being the vast majority of tumors met in this field [4].

Every case of extracranial meningiomas provides a description of the histological characteristics; they are divided into different subtypes, in consideration of the most predominant cellular morphology, including meningothelial (syncytial), fibrous (fibroblastic), psammomatous, angiomatous (angioblastic), and transitional (mixed) [3]. The risk of extracranial meningiomas increases with age, also being more susceptible in women than men. Regarding the therapeutical approaches, total surgical resection is the primary treatment of choice for extracranial meningiomas [5].

## Case presentation

**Figure 1:**
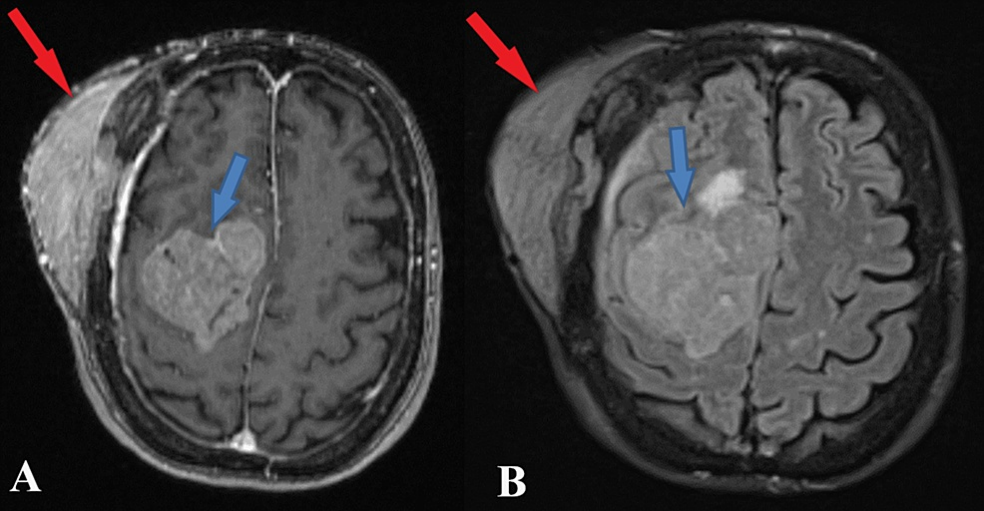
Preoperative MRI T1 and T2 sequence The axial section of T1 sequence (A) and axial section of T2 sequence (B) both depict a giant intracranial (blue arrows) and extracranial (red arrows) meningioma with significant osteolysis of the skull after extensive extracranial invasion.

**Figure 2:**
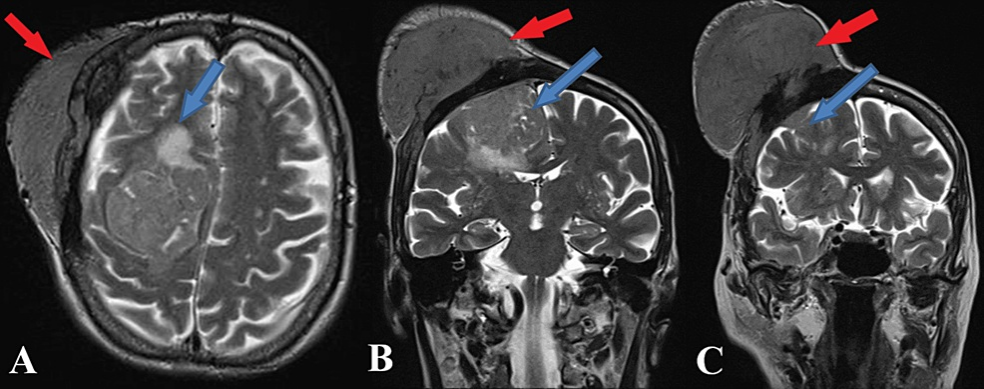
Preoperative MRI T2 FLAIR sequence Axial section (A). Coronal section (B,C). The arrows point to a giant intracranial (blue arrows) and extracranial (red arrows) meningioma with significant osteolysis of the skull after extensive extracranial invasion.

This is a case of a 69-year-old patient with a known history of cardio-respiratory disorders, such as atrial fibrillation with medium rhythm and bilateral bronchopneumonia. Neurological examination reveals left hemiparesis, intracranial hypertension syndrome, cerebral edema, and cerebrospinal fluid (CSF) fistula of the frontal lesion. Following morphopathological analysis, we suspect that the patient was experiencing a meningioma en plaque (MEP), which is an infiltration at the level of the dura and sometimes invades the bone with intraosseous tumor growth, leading to significant hyperostosis. The patient also shows osteolytic change of the skull, an element found in MEP (Figures 1-2).

**Figure 3:**
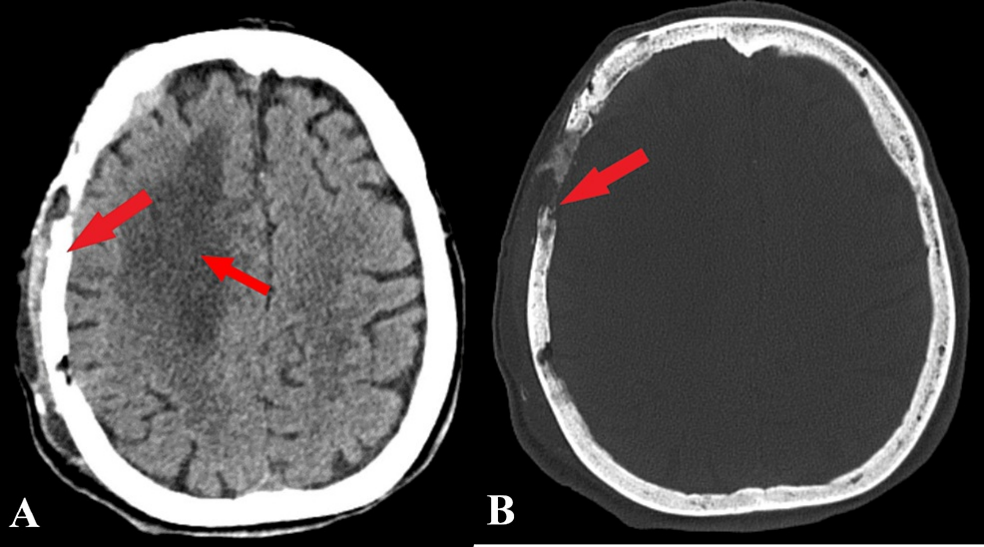
Postoperative CT CT tissue window (A); the red arrows indicate the gross total tumor resection, while the CT bone window (B) shows the postoperative still skull lysis (pointed by the red arrow) due to extracranial invasion.

After surgical intervention, achieving gross total resection (Figure 3), the patient suffered cardiorespiratory arrest of cardiac cause, followed by acute respiratory failure and ventilator prosthesis.

The patient required intubation and mechanical ventilation over 96 hours, presenting NYHA II heart failure, acute hemodynamic failure, acid-base balance disorders, and upper gastrointestinal hemorrhage.

## Discussion

As stated before, histology defines five patterns of these tumors, of which syncytial (meningothelial) ones are most prevalent among the primary extracranial meningiomas [3]. A neoplasm composed of spindle-shaped cells with whorls, without any indication of atypia, suggests a meningothelial origin [6]. Along with this subtype, psammomatous meningioma, a densely calcified tumor characterized by the presence of numerous psammoma bodies, is most common in vertebral disorders.

Fibrous meningiomas are identified by extended spindle-shaped tumor cells with narrow rod-shaped nuclei that are set in a collagenous or reticulum-rich environment. Compared to meningothelial subtypes, these have fewer whorls, and psammoma bodies are sporadically found [7]. Typically, transitional meningiomas display meningothelial cells organized in bundles of variable length, exhibiting a mix of specific fibroblastic appearance, syncytial arrangements, epithelioid cells, and consistently occurring whorls. Faint cytoplasmic boundaries and occasional intranuclear inclusion bodies are usually described [8].

A rare subtype of meningiomas, comprising only 2.1% of them, is represented by the angiomatous one. Such tumors are described by an abundance of blood vessels amidst meningothelial areas. They are classified as WHO grade I tumors without cellular atypia or anaplasia [9,10].

Overall, meningiomas are classified into three grades (1-3) based on their histological and molecular features. Grade 1 subtype displays less aggressive characteristics, accordingly, while second and third grade proves to be more concerning. Thus, grade 2 tumors exhibit increased mitotic figures, invasion of the brain tissue, and specific histological subtypes (choroid or clear cell kinds), while grade 3 surpasses the prior ones, describing hallmarks such as sarcoma, carcinoma, or melanoma-like appearances, along with telomerase reverse transcriptase (TERT) promoter mutation [11].

Among the various types of meningiomas that are being found within a mass, MEP is quite uncommon, and only a few reported cases have presented tumors that merge both the extradural and MEP types [12]. They display a ‘carpet-like’ infiltration of the adjacent bone, accompanied by extensive hyperostosis and dural thickening [13]. Typically, extradural spinal meningiomas arise from nerve roots, where the dura is thinner, thus making it easier to migrate into the extradural space. More often, the meningioma appears as a round type, while the en plaque type grows along the dura mater in sheet-like structures [11].

While studying the literature on extracranial meningiomas, we have delved into many studies that unveiled significant contributions to the field, many of them being elaborated in Table 1.

**Table 1:** Illustrates a compilation of studies about extracranial meningiomas and their features N = number of patients; F/M = female/male patients; GTR = total growth resection; STR = subtotal growth resection; PR = partial resection; WHO Gr = histological grade according to the World Health Organization; R = recurrences rate.

First author & year	N	Sex, N (%)		Extent of removal, N (%)			Surgical approach	Postoperative complications, No. of patients (%)		
		F	M	GTR	STR	PR		Histological aspect	Patients’ follow-up	
Jian et al. 2005 [14]	9	4 (45%)	5 (55%)	9 (100%)	0 (0%)	0 (0%)	Barbosa approach	Neurofibrosarcoma: 1 (11%); Adenoid cystic carcinoma: 2 (22%); Inflammatory pseudotumor: 2 (22%) Ameloblastoma: 1 (11%); Mucoepidermoid carcinoma: 2 (22%); Extracranial meningioma: 1 (11%).	R: 1 (11%);	
Rushing et al. 2009 [1]	146	74 (50.6%)	72 (49.31%)	100 (68.49%)	36 (24.65%)	10 (6.84%)	Wide local excision	WHO Gr I: 128 (87.67%) WHO Gr II: 14 (9.58%) WHO Gr III: 4 (2.73%)	R: 26 (23,6%); Mortality: 13 (8.9%)	
Aiyer et al. 2012 [3]	3	3 (100%)	0 (0%)	100 (0%)	0 (0%)	0 (0%)	Left lateral rhinotomy approach	Two types of cells with rounded or oral and elongated nuclei in the spindle type.	R: 1 (33%)	
Possanzini et al. 2012 [2]	3	3 (100%)	0 (0%)	3 (100%)	0 (0%)	0 (0%)	Wide local excision	Nodular cell clusters with regular nuclei; Neoplastic growth comprised of lobules of neoplastic cells and bound by fusocellular cells; enhanced vortex and syncytial structures; Fusocellular proliferation involving chorion, intermixed with fibrous regions and locally infiltrating trabecular bone tissue.	R: 0 (0%)	
Jang et al. 2014 [15]	1	1 (100%)	0 (0%)	1 (100%)	0 (0%)	0 (0%)	Supraorbital eyebrown approach	Various histological types are found as meningioma en plaque (MEP)	R: 0 (0%)	
Behling et al. 2020 [16]	1517	1115 (73.5%)	402 (26.5%)	366 (24.8%)	411 (27.8%)	701 (47.4%)	Wide local excision, with fluorescence techniques	WHO Gr I: 1281 (84.4%) WHO Gr II: 232 (15.3%) WHO Gr III: 4 (0.3%)	R: 149 females (38.1%); 93 men (51.5%)	
He et al. 2022 [17]	53	40 (75.4%)	13 (24.6%)	33 (62.3%)	20 (37.7%)	0 (0%)	Orbitofrontal approach: 16 (30.18%); Pterional approach: 15 (28.3%); Orbital zigomatic approach: 18 (35.84%); Endoscopic endonasal approach: 3 (5.6%)	WHO Gr I: 45 (84.9%) WHO Gr II: 8 (15.1%)	R: 18 (34.9%); Increased visual acuity: 18 (34.9%); Lower visual acuity: 2 (3.77%)	

From the data presented, we have shown that there are multiple surgical strategies, such as wide local excision and supraorbital eyebrow approach, which is a minimally invasive technique that offers wide access to the anterior skull base region and parasellar area through a subfrontal corridor. The use of neuroendoscopy allows one to extend the approach further to the pituitary fossa, the anterior third ventricle, the interpeduncular cistern, the anterior and medial temporal lobe, and the middle fossa. The supraorbital approach involves a limited skin incision, with minimal soft-tissue dissection and a small craniotomy, thus carrying relatively low approach-related morbidity. Using this technique, the risk of recurrence was 0% [18].

Table 1 highlights the relationship between the degree of resection of the tumor and the risk of recurrence so that patients in whom total growth resection (GTR) was possible have a much lower risk of recurrence than those in whom STR or PR could be performed. In the cases presented, in patients in whom total tumor resection was possible, recurrence was 0%. Most recurrences occurred about five years after surgery [19].

From the cases presented in the table, we have highlighted the greater exposure that women experience, compared to men, in the case of this pathology, as women are more likely to develop this disease. Additionally, older ages, over 65 years, favor the appearance of this disease, as it is in our case.

From the data presented, we have shown that there are multiple surgical strategies, highlighting the relationship between the degree of resection and the risk of recurrences so that patients in whom GTR was possible have a much lower risk than those in whom subtotal growth resection (STR) or partial resection (PR) could be performed. In the cases presented, where total tumor resection was possible, the recurrence rate was 0%, most of them occurring about five years after surgery. We also took into account the histological aspect, useful in the diagnosis of this disease [20].

## Conclusions

In wrapping up our discussion about this patient’s fascinating medical case, we have unraveled significant aspects regarding the pathology of meningiomas. Our female patient possesses all the factors that make insightful contributions to the condition she experienced as follows: her advanced age, her clinical picture through the neurological deficits she had, and the tumor’s location, along with the osteolytic changes. The surgical approach allowed the resection of the entire tumoral process, thus preventing any recurrence and ensuring the patient’s wellness.
